# Catalyst-Driven Improvements in Conventional Methods for Imine-Linked Covalent Organic Frameworks

**DOI:** 10.3390/molecules30142969

**Published:** 2025-07-15

**Authors:** Maziar Jafari, Zhiyuan Peng, Ali Samie, Faezeh Taghavi, Amir Khojastehnezhad, Mohamed Siaj

**Affiliations:** 1 Department of Chemistry, University of Quebec at Montreal, Montreal, QC H3C 3P8, Canada; jafari.maziar@courrier.uqam.ca; 2 Department of Chemical Engineering and Biotechnological Engineering, Faculty of Engineering, Université de Sherbrooke, Sherbrooke, QC J1K 2R1, Canada; peng.zhiyuan@courrier.uqam.ca (Z.P.); mohamed.siaj@usherbrooke.ca (M.S.); 3 Department of Medicinal Chemistry, School of Pharmacy, Mashhad University of Medical Sciences, Mashhad, Iran; alisamie.chemistry@gmail.com; 4 Department of Chemistry, Faculty of Science, Ferdowsi University of Mashhad, Mashhad, Iran; taghavi_negin@yahoo.com

**Keywords:** COFs, covalent organic frameworks, porous materials, catalyst, polymers

## Abstract

Imine-linked covalent organic frameworks (COFs) have attracted considerable interest in recent years because they can form strong and reversible covalent bonds, enabling the development of highly ordered crystalline structures. This reversibility is crucial in correcting structural defects during the crystallization process, which requires sufficient time to proceed. This review critically examines the advancements in synthetic strategies for these valuable materials, focusing on catalytic versus conventional approaches. Traditional methods for synthesizing imine-linked COFs often involve harsh reaction conditions and prolonged reaction times, which can limit the scalability and environmental sustainability of these frameworks. In contrast, catalytic approaches offer more efficient pathways, enabling shorter reaction times, milder reaction conditions, and higher yields. This article elucidates the key differences between these methodologies and examines the impact of reduced reaction times and milder conditions on the crystallinity and porosity of COFs. By comparing the catalytic and conventional synthesis routes, this review aims to provide a comprehensive understanding of the advantages and limitations of each approach, offering insights into the optimal strategies for the development of high-performance COFs.

## 1. Introduction

Imine-linked covalent organic frameworks (COFs) are important due to their unique structural properties, such as high stability, porosity, and tunable functionality. These frameworks offer significant advantages in various applications, including catalysis [1,2,3,4], drug delivery and biosensors [5,6,7], gas and energy storage [8,9,10,11], and separation and purification [12,13,14,15,16,17]. This is primarily due to their ability to form strong, reversible bonds that facilitate the creation of highly ordered crystalline materials. The imine linkage also provides chemical robustness, making these COFs suitable for use in harsh environments, while their design flexibility allows for the incorporation of different functional groups, enhancing their utility in fields ranging from environmental remediation to drug delivery.

The synthesis of COFs has witnessed an expansive array of methods, each tailored to achieve specific structures and properties [18,19,20], which involves the formation of covalent bonds through reversible reactions between carefully chosen organic building units [21]. This synthetic versatility has led to the exploration of numerous variations in building units, linkages, and functionalities, giving rise to an extensive library of COFs with distinct properties [22,23,24]. While the potential applications of COFs are vast and promising, challenges exist, and ongoing research efforts are focused on addressing them. The scalability and reproducibility of COF synthesis methods are areas of active investigation, with researchers working to develop efficient and cost-effective approaches for large-scale production. Additionally, understanding the fundamental principles governing the properties of COFs is essential for advancing their design and application [20,25,26].

The conventional synthesis of COFs predominantly relies on solvothermal or hydrothermal techniques, conducted within sealed high-pressure tubes. In this method, organic precursors undergo polycondensation in the presence of solvents under elevated temperatures [27]. Specifically, starting materials, solvents, and traditional acid catalysts such as CH_3_CO_2_H or CF_3_CO_2_H are combined. The tube is then sealed to retain the generated water molecules, thereby ensuring the reversible reaction conditions necessary for crystallization. The reaction vessel is typically subjected to specific temperatures in an oven or oil bath for several days (e.g., 3–7 days) to produce high-quality COFs [28,29,30,31]. Despite their widespread use, numerous variables profoundly affect COF crystallinity during synthesis, including catalyst loading, solvent volume ratios, reaction duration, pressure, and temperature. Achieving optimal COF quality often demands arduous optimization, particularly extensive solvent screening, which represents a substantial bottleneck in COF development. Moreover, even when optimized conditions are established, the inherent sensitivity of COF formation to synthetic parameters frequently leads to significant batch-to-batch inconsistencies. Thus, the pursuit of novel synthetic strategies for reproducibly generating high-quality COFs is both a critical need and a formidable challenge.

Acetic acid, a versatile and weak monoprotic acid (pKa = 4.76), is widely utilized in organic synthesis due to its ability to effectively protonate substrates, thereby facilitating various organic transformations. Its relatively non-toxic nature and ease of handling make it the most commonly employed catalyst for constructing imine-based COFs. In 2009, Yaghi et al. highlighted the critical role of acetic acid in the synthesis of crystalline materials, particularly by preparing the highly crystalline imine-based 3D COF-300 [32]. This COF exhibited exceptional crystallinity, porosity, stability, and structural resilience in various organic solvents. The material and its analogs have since become essential for investigating structure–property relationships in COFs. Following this, acetic acid demonstrated significant versatility with diverse building blocks and reaction systems, making it the preferred choice for synthesizing various COFs.

Despite its widespread adoption [33,34,35], the conventional use of acetic acid as a catalyst for imine-linked COFs has faced increasing criticism. This is particularly due to its limited universality, with reaction conditions and times being harsh and lengthy [31,36]. This limitation means that only a narrow range of imine-linked COFs can be reliably synthesized with a high crystallinity and porosity using this method. Consequently, many researchers have resorted to alternative physical activation techniques, such as the application of light [37], sonication [38], microwave irradiation [39], or electron beam irradiation [40].

The catalyst’s intrinsic role in COF synthesis is paramount, directly influencing reaction efficiency, the resulting COF product quality, and overall yield. Catalysts facilitate the crucial reversible bonding between monomers and organic linkers. A growing body of literature demonstrates that innovative catalytic strategies can not only optimize reaction times and conditions but also offer tunable control over COF morphology, crystallinity, and porosity [41,42,43]. Crucially, these advanced catalytic approaches often circumvent the need for additional mechanical, acoustic, or photonic energy stimuli, simplifying synthetic procedures and potentially improving scalability.

**Figure 1:**
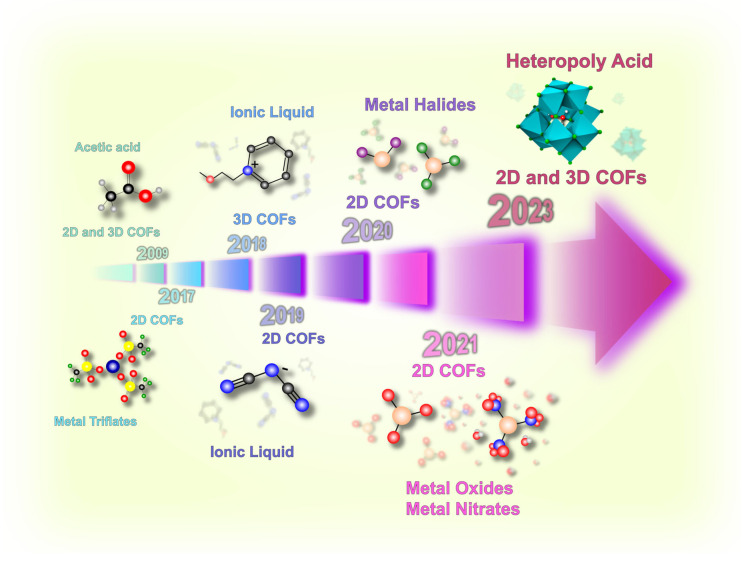
Chronological evolution of catalyst strategies for the synthesis of imine-linked COFs. Early approaches primarily relied on Brønsted acid catalysis under solvothermal conditions, transitioning to more recent advancements including ambient aqueous synthesis, often acid-catalyzed, and the exploration of novel Lewis acid catalysts.

In this review, we comprehensively summarize the recent advancements in catalytic strategies for imine-linked COFs, focusing on the use of metal triflates, ionic liquids (ILs), metal oxides, metal halides, metal nitrates, and heteropoly acids. These catalyst classes have consistently demonstrated the ability to produce high yields of highly crystalline and porous imine-linked COFs under mild conditions, typically involving low temperatures, shorter reaction times, and remarkably low catalyst quantities. The chronological development and application of these strategies within the context of imine-linked COFs are visually represented in Figure 1. The resulting imine-linked COFs synthesized via these catalytic pathways have found broad applicability across diverse fields, including optoelectronics, selective gas absorption, heterogeneous catalysis, solar cells, and supercapacitors. Although several review articles on COF synthesis have been published to date, they generally address broader synthetic methodologies or application domains, and none have focused specifically on the catalytic synthesis of imine-linked COFs as the central theme [44,45]. Furthermore, we extend our discussion to the potential applicability of these robust catalytic strategies to the synthesis of other important COF linkages, such as azine-, fused-ring-, hydrazone-, imide-, ketoenamine-, olefin-, triazine-, and tetrahydroquinoline-linked COFs.

## 2. Catalytic Synthesis of Imine-Linked COFs

**Scheme 1:**
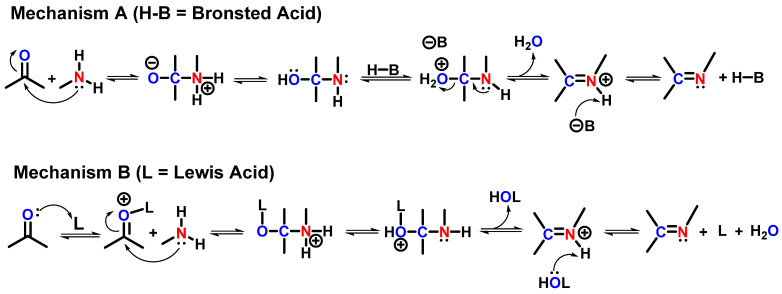
Catalytic mechanisms for COF formation via Schiff base polycondensation reaction.

Over the past decade, significant progress has been made in diversifying catalytic strategies for the synthesis of imine-linked COFs, moving beyond traditional Brønsted acid catalysis toward more efficient and environmentally friendly alternatives. Acetic acid remains the most commonly used catalyst due to its simplicity, availability, and ability to promote reversible imine bond formation. However, its application often requires harsh solvothermal conditions, extended reaction times, and intensive solvent screening, factors that limit scalability and reproducibility. In response, recent efforts have focused on developing novel catalytic systems such as Lewis acids, metal triflates, metal oxides, metal nitrates, metal halides, and heteropoly acids which can offer milder conditions, improved yields, and superior control over crystallinity and porosity. Two representative mechanisms illustrate this shift: in the conventional Brønsted acid-catalyzed method, the nucleophilic lone pair on the amine attacks the carbonyl group, leading to the formation of a hemiaminal intermediate, which then undergoes dehydration to produce the imine linkage. Throughout this transformation, Brønsted acid catalysts like acetic acid assist by stabilizing the intermediate and promoting the condensation step (Scheme 1, Mechanism A); in contrast, Lewis acid catalysts, such as metal triflates or halides, coordinate with carbonyl or imine nitrogen atoms to accelerate imine formation, enabling synthesis under milder conditions with significantly reduced reaction times (Scheme 1, Mechanism B). These advancements not only enhance the structural quality and functional tunability of COFs but also represent a crucial step toward sustainable and scalable production for diverse technological applications.

### 2.1. Metal Triflates

**Figure 2:**
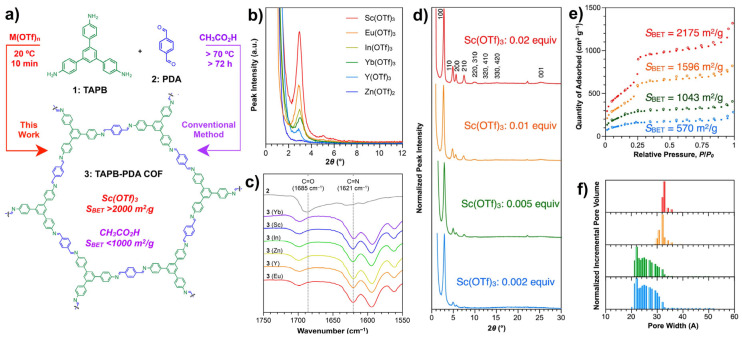
Synthesis of imine-linked COFs in the presence of acetic acid and Sc(OTf)_3_ (catalytic approach) (**a**). PXRD patterns of TAPB-PDA COFs synthesized with In(OTf)_3_, Sc(OTf)_3_, Yb(OTf)_3_, Y(OTf)_3_, Eu(OTf)_3_, and Zn(OTf)_3_ as catalysts (**b**). FT-IR spectra confirming the successful polycondensation for each synthesized TAPB-PDA COF (**c**). PXRD patterns illustrating the effect of catalyst loading on COF crystallinity (**d**). BET surface area (**e**) and pore size distribution curves (**f**), both demonstrating the effect of catalyst loading. Reprinted with permission from ref. [79]. Copyright 2017, American Chemical Society.

Imine-linked COFs have raised recent interest because of the variety of condensable monomers and their superior oxidative and hydrolytic stability [28,29,31,46,47,48,49,50,51,52,53,54,55,56,57]. For synthesizing imine-linked COFs, acetic acid (CH_3_CO_2_H) and acetic acid derivatives are trivially used to catalyze imine formation and exchange after condensing polyfunctional aldehydes and amines at elevated temperatures in a mixture of organic solvents [28,29,31,37,38,39,40,50,54,56,58,59,60]. Although effective in forming crystalline, uniformly porous, and moderate-surface area products with numerous design monomers, the reaction conditions are harsh and lengthy [28,29,50,56]. It has been recently discovered that the rate-limiting step in imine-linked COF formation is the transimination step, and for this, acetic acids are not particularly good catalysts [61,62,63]. Metal triflates are Lewis acidic metal salts derived from metal hydroxides and triflic acid, known for their stability and solubility in organic solvents. They are widely used as catalysts in organic synthesis, offering high reactivity and selectivity in a variety of chemical transformations, including carbon–carbon bond formation and rearrangement reactions [62,63,64,65,66,67,68,69,70,71,72,73,74,75,76,77,78]. Instead of using acetic acid, Matsumoto et al. tested 2D COF synthesis using metal triflate Lewis acids as catalysts [79] because of their good imine formation and transimination properties while having a high water tolerance and functional group compatibility (Figure 2a) [61,62,63,73]. They showed that indium, scandium, ytterbium, yttrium, europium, and zinc triflates (In(OTf)_3_, Sc(OTf)_3_, Yb(OTf)_3_, Y(OTf)_3_, Eu(OTf)_3_, and Zn(OTf)_3_) all yielded over 95% highly crystalline TAPB-PDA COF products after a 72 h reaction time at room temperature, at extremely low loadings of 0.0001 equiv. relative to the amine and aldehyde functional groups of tris(amine) (TAPB) and terephthaldehyde (PDA), respectively (Figure 2b). However, activation with supercritical CO_2_ was conditional to the high crystallinity, as activation by solvent evaporation under vacuum provided variable crystallinity due to pore collapse or partial exfoliation during evaporation disrupting interlayer stacking [79]. Precipitates were observed in as little as 1 min and a crystalline network could be isolated in as little as 10 min. Using acetic acid at these low concentrations presented no product even after 14 days at 90 °C, highlighting the potency of metal triflates as effective catalysts for the formation of imine-linked COFs. FT-IR confirmed the conversion from aldehydes to imines by the disappearance of the carbonyl stretch at 1685 cm^−1^ and the prominent peak surging at 1620 cm^−1^ (Figure 2c). Among the metal triflates, COFs made with scandium triflate (Sc(OTf)_3_) presented remarkably more crystalline structures, explained by the smaller ionic radii of Sc ions acting as a more effective catalyst (Figure 2b) [62,63]. With Sc(OTf)_3_, optimal crystallinity was noted in as little as 10 min between 0.002 and 0.02 equiv. Higher loadings showed broader peaks on the diffractogram, consistent with previous studies (Figure 2d) [63]. A higher surface area (2175 m^2^ g^−1^) with a narrower pore size distribution of 3.4 nm was measured using 0.02 equiv., compared with using a lesser 0.01 equivalent (1569 m^2^ g^−1^) (Figure 2e,f). Further increasing the catalyst loading also decreased the surface areas and broadened the pore size distributions. The authors demonstrated the generality and the functional group tolerance of this catalyst by making a crystalline TAPB-BPDA 2D COF with a surface area of 1235 m^2^ g^−1^. Comparatively, under typical conditions with acetic acid (20 equiv., 70 °C, 3 days), the TAPB-BPDA yield was amorphous with a low surface area (54 m^2^ g^−1^). A TAPB-TIDA thienoisoindigo derivative 2D COF, useful for optoelectronic applications, was synthesized using Sc(OTf)_3_. The product was crystalline with a moderate surface area (692 m^2^ g^−1^). Surprisingly, the acetic acid-catalyzed TAPB-TIDA 2D COF was isolated with a higher crystallinity and porosity (1675 m^2^ g^−1^). Another example of a uniform, crystalline, and high-surface area imine-linked 2D COF was reported by Dichtel’s group [80]. They also synthesized this COF via the condensation of TAPB and PDA, catalyzed by Sc(OTf)_3_. The proven effectiveness of this catalyst in the synthesis of imine-linked COFs is evidenced by its widespread adoption in the recent literature, where it has consistently demonstrated a reliable performance in promoting high crystallinity, improved porosity, and efficient reaction conditions across a range of studies [81,82,83,84]. In a different approach, Li et al. constructed “smart COF capsules” for enzyme encapsulation [85]. Their method utilized ZIF-90 as a degradable MOF template and COF-42, a hydrazone-linked COF, as the scaffold. The room-temperature condensation of 1,3,5-triformylbenzene (TB) and 2,5-bis(ethoxy)-terephthalohydrazide (BETH) was also catalyzed by Sc(OTf)_3_ in this work. A Chinese research group demonstrated the synthesis of highly crystalline and porous tetrahydroquinoline-linked COFs within three days, using Sc(OTf)_3_ and Yb(OTf)_3_ as catalysts [86]. Separately, Feng et al. successfully synthesized fused-ring linked COFs at 120 °C over a five-day period, also employing Sc(OTf)_3_ as a catalyst [87]. Interestingly, when the same monomers were used with acetic acid as a catalyst, the corresponding imine-linked COF was formed. These results highlight the broad applicability of metal triflates, particularly the Sc(OTf)_3_-catalyzed synthesis of various COF architectures.

### 2.2. Ionic Liquids

**Figure 3:**
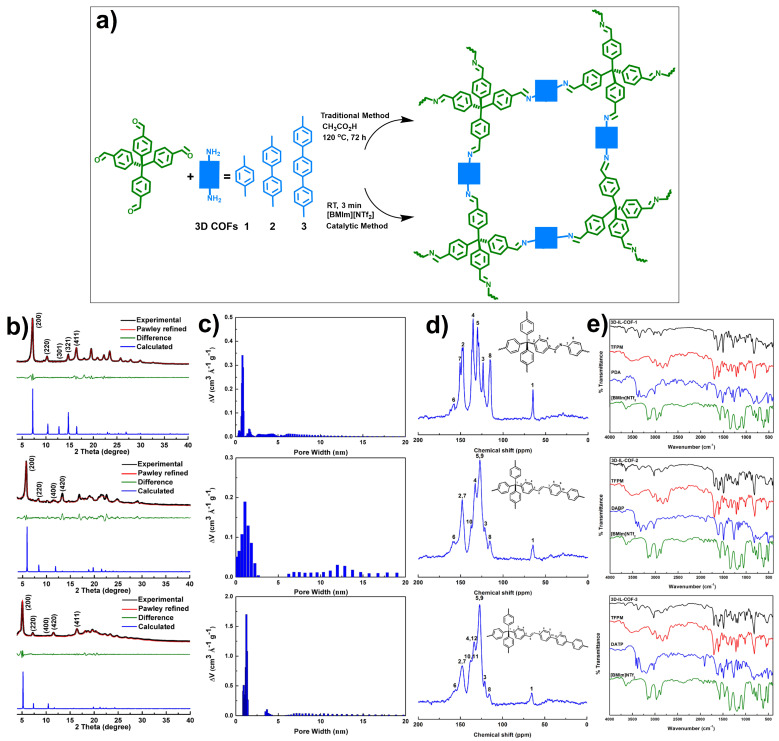
Synthesis of imine-linked 3D COFs (3D-IL-COF-1, 3D-IL-COF-2, and 3D-IL-COF-3) in the presence of acetic acid and IL (catalytic approach) (**a**). PXRD patterns (**b**), BET-calculated pore size distribution curves (**c**), ^13^C cross-polarization/magic angle spinning (CP/MAS) NMR spectra (**d**), and FT-IR spectra of 3D-IL-COF-1, 3D-IL-COF-2, and 3D-IL-COF-3 (**e**). Reprinted with permission from ref. [91]. Copyright 2018, American Chemical Society.

Ionic liquids (ILs) are salts that remain in the liquid state at or near room temperature and exhibit unique properties such as low volatility, high thermal stability, and tunable solvation behavior, making them attractive for green chemistry applications [88]. Their wide electrochemical window and ability to dissolve a variety of compounds enable their use in catalysis, electrochemistry, and material synthesis [89]. Recent advancements also highlight their role as designer solvents for sustainable chemical processes [90]. As for 2D COFs, 3D COFs are classically prepared by solvothermal synthesis methods at elevated temperatures and pressures in sealed tubes, demanding complex operations and possibly releasing volatile organic compounds (VOCs). An alternate synthesis method applicable to the large-scale green production of 3D COFs using ILs as catalysts was proposed by Guan et al. They were inspired by the use of ILs for the synthesis of crystalline materials [91], such as zeolites metal–organic frameworks (MOFs) [92,93,94] and porous polymers [95,96]. The authors proposed the first basic ionothermal synthesis of 3D COFs in ambient conditions with multifold interpenetrated diamondoid (dia) nets [97]. This synthesis method offered advantages, such as fast yield times, catalyst recycling, and even selective gas separation properties. The IL 1-butyl-3-methylimidazolium bis((trifluoromethyl)sulfonyl)imide ([BMIm][NTf_2_]) was both the solvent and the catalyst for the Schiff base reactions between tetrakis(4-formylphenyl)-methane (TFPM) and *p*-phenylenediamine (PDA), 4,4′-diaminobiphenyl (DABP) or 4,4″-diamino-*p*-terphenyl (DATP) monomers, forming highly crystalline 3D COFs (3D-IL-COF-1, 3D-IL-COF-2, and 3D-IL-COF-3, respectively) of similar chemistry with increasing pore size and yielding 3D-IL-COFs in as little as 3 min (Figure 3a). These reaction times are considerably faster than the 3–7 days needed for traditional solvothermal methods [98,99]. The sharp and intense crystalline peaks in PXRD patterns enabled lattice parameter calculations for each of the 3D COFs, and additional unexpected peaks at higher diffraction angles were ascribed to guest ILs in the micropores (Figure 3b) [32,100]. The 11-fold interpenetrated dia of 3D-IL-COF-3 is the most interpenetrated framework for COFs reported to date. The BET surface areas were 517 m^2^ g^−1^, 653 m^2^ g^−1^, and 870 m^2^ g^−1^ for 3D-IL-COFs-1, -2, and -3, respectively, and the measured pore widths of 0.83, 1.07, and 1.24 nm closely agreed with calculations using nonlocal density function theory (NLDFT) (Figure 3c). Successful synthesis was confirmed by FT-IR by observing the C=N stretching peak from 1622 to 1624 cm^−1^ and by the presence of the C=N bond with ^13^C (CP/MAS) NMR spectra at 157–159 ppm (Figure 3d,e). Moreover, the detection of IR peaks corresponding to the IL confirmed successful inclusion within the 3D COFs’ structure. Thermogravimetric analyses showed that the 3D COFs had a high thermal tolerance up to 450 °C and 8–12% IL retention in pores. Interestingly, all three COFs had a remarkably higher affinity for CO_2_ absorption than for CH_4_ and N_2_. This is attributed to the selectivity between the ILs and CO_2_, which is useful for potential environmental applications.

**Figure 4:**
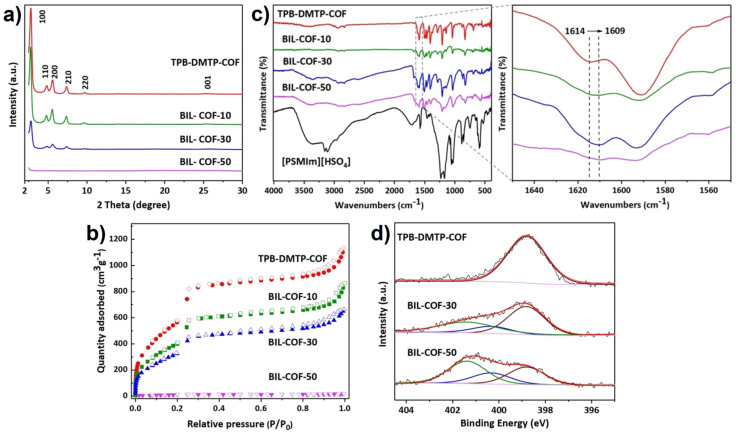
PXRD patterns (**a**), N_2_ adsorption–desorption isotherms (**b**), FT-IR spectra (**c**), and XPS spectra of 2D TPB-DMTP-COF, BIL-COF-10, BIL-COF-20, and BIL-COF-50 (**d**). Reprinted with permission from ref. [101]. Copyright 2019, The Royal Society of Chemistry.

Du et al. effectively synthesized a 2D imine-linked COF from triphenylbenzene (TPB) and dimethoxyterephthaldehyde (DMTP) using the Brønsted acid ionic liquid (BIL) 1-methyl-3-(3-sulfopropyl)-1H-imidazol-3-ium hydrosulfate ([PSMIm][HSO_4_]) [101]. This BIL served a dual role, acting as both the catalyst and solvent and as a guest molecule within the COF framework. The authors demonstrated that the COF retained its crystallinity even with high BIL loadings, ranging from 10 to 30 g/L (Figure 4a). However, porosity was inversely affected by increasing BIL content. The BET surface area decreased significantly from 2231 m^2^ g^−1^ for the pristine TPB-DMTP-COF to 1550 and 1266 m^2^ g^−1^ for BIL-COF-10 and BIL-COF-30, respectively (Figure 4b). Concurrently, by using IL, the pore sizes narrowed (from 2.7 nm to 2.2 nm), and the pore volumes decreased (from 1.75 cm^3^ g^−1^ to 1.03 cm^3^ g^−1^) for pristine TPB-DMTP-COF and BIL-COF-30, respectively, showing that the cavities of the COF were partially occupied by ILs. A very high loading of 50 g/L resulted in an amorphous material with a dramatic loss of porosity. Thermogravimetric analysis revealed a two-step decomposition pattern upon BIL inclusion, while FT-IR spectroscopy confirmed successful polycondensation by the disappearance of the C=O stretching vibration (from TAPB) and N-H stretching (from DMTP), alongside the appearance of the C=N bond at approximately 1614 cm^−1^. A noticeable red shift in the C=N bond suggested the formation of hydrogen bonds between the COF backbone and the BIL (C=N⋅⋅⋅H formations) (Figure 4c), a finding further corroborated by a positive N1s peak shift in the XPS spectra (Figure 4d). Impressively, the BIL-COF-30 exhibited an outstanding catalytic performance, achieving a 97% conversion from sorbitan to isosorbide with minimal byproduct generation (only 3%). Furthermore, this catalyst demonstrated excellent reusability, maintaining over 90% of its initial activity for at least five consecutive cycles. Gao et al. successfully synthesized mesoporous and microporous imine-linked 2D COFs in 1-butyl-3-methylimidazolium (bmim^+^) ILs, systematically investigating the impact of the IL counter anion on COF formation [102]. Extending this strategy to other types of COF linkers, Guan et al. demonstrated the formation of 3D-HNU5, an azine-linked COF, with an interpenetrated diamond topology [103]. This was achieved by reacting tetrakis(4-formylphenyl)methane (TFPM) with hydrazine in [Bmim][Tf_2_N] ILs. The resulting COF exhibited enhanced selectivity for CO_2_ over N_2_ absorption and efficient catalytic activity for the cycloaddition of propargylic alcohols with CO_2_ into carbonates under mild conditions. Further showcasing the utility of ILs, Qiu et al. assembled *β*-ketoenamine-linked hierarchically porous COFs (HP-COFs) from 1,3,5-triformylphloroglucinol (Tp) and 4,4’-azobenzenediamine (Azo) [104]. This synthesis, conducted in 1-alkyl-3-methylimidazolium tetrafluoroborate ILs ([C_n_mim][BF_4_], *n* = 4, 6, 10), yielded HP-COFs with a high crystallinity and tunable porosity by adjusting the alkyl chain length of the IL. Remarkably, these HP-COFs were assembled under mild conditions (50 °C within 12 h) and displayed significant catalytic activity for C-C bond formation, particularly for larger molecules. In a distinct approach, Dong et al. fabricated 2D ketoenamine-linked COFs using a room-temperature IL as a solvent, which is notable because the IL was entirely removed after COF assembly, serving exclusively as a solvent and catalyst [105]. Beyond synthesis, Xin et al. showcased an early example of directly incorporating an IL within the nanopores of a 3D COF [100].

### 2.3. Metal Oxides

**Figure 5:**
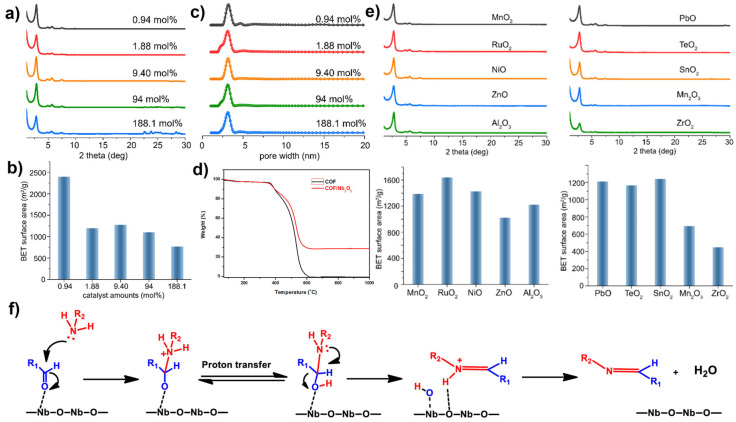
PXRD pattern (**a**), BET surface area (**b**), and pore size distribution (**c**) of TAPB-OMePDA COF synthesized using Nb_2_O_5_ as a catalyst with different catalyst loading. TGA curve showing the retention of catalyst within the final TAPB-OMePDA COF product (**d**). PXRD patterns and BET surface areas of TAPB-OMePDA COFs synthesized with various metal oxide catalysts (**e**). Author-proposed Schiff base reaction mechanism catalyzed by Nb_2_O_5_ (**f**). Reprinted with permission from ref. [116]. Copyright 2021, American Chemical Society.

**Figure 6:**
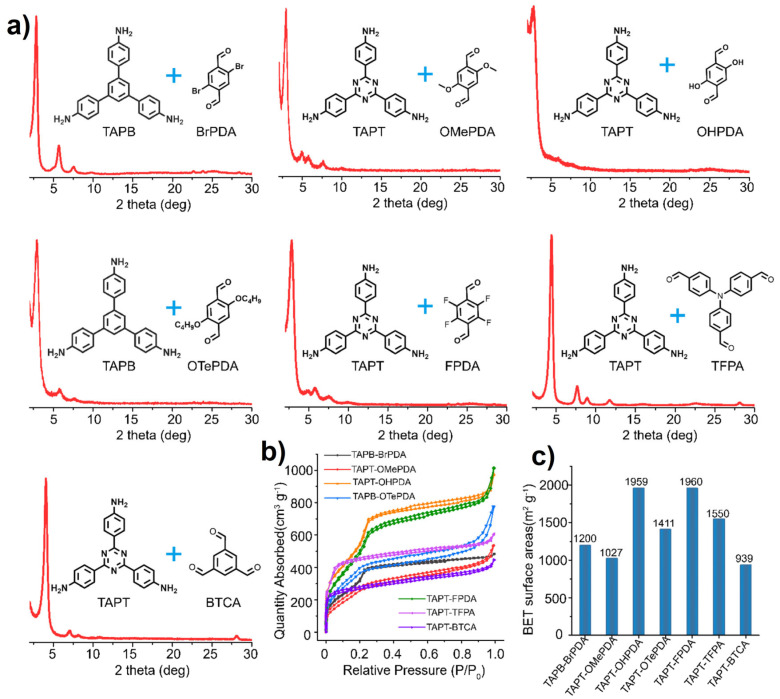
PXRD patterns (**a**), N_2_ adsorption–desorption isotherms (**b**), and BET surface areas (**c**) of imine-linked COFs with different starting materials and with the use of Nb_2_O_5_. Reprinted with permission from ref. [116]. Copyright 2021, American Chemical Society.

Other types of imine–COF composites are COFs integrating metal oxides [106], perovskites [107], or metal particles [108]. These COFs are crystalline, porous, and have high surface areas, in addition to having the functional properties from the enclosed materials [109]. These COF composites find utility in chemical absorption and magnetic resonance imaging [109], photovoltaics and photocatalysts [106], lasers [110], and X-ray detectors [111]. Methods preparing metal oxide/COF composites require complex multistep protocols [112,113]. On the other hand, simple physical mixtures are ineffective due to practical limitations [109]. Alternatives propose the growth of COFs on the metal oxide (MO) particle or substrate surface [114,115]. Zhu et al. introduced a simple one-pot reaction leveraging the strong Lewis acidity of MOs, including Nb_2_O_5_, RuO_2_, TiO_2_, Mn_2_O_3_, and Fe_3_O_4_, to produce MO/COF composites rapidly in mild conditions with minimal loading [116]. Using 0.94 to 188.1 mol% relative to amine monomers, Nb_2_O_5_ was used as a catalyst to synthesize (TAPB-OMePDA) COF from 1,3,5-tris(4-aminophenyl)benzene (TAPB)−2,5-dimethoxyterephthaldehyde (OMePDA) at 120 °C in 3 days. Although all Nb_2_O_5_/COFs had excellent crystallinity according to PXRD (Figure 5a), the lowest catalyst loading reached a high surface area of 2405 m^2^ g^−1^, even without supercritical CO_2_ activation, which is among the highest reported for a 2D COF (Figure 5b) [59,79,107]. At 188.1 mol% catalyst loading, the COF surface area was predictably lower and calculated to be 774 m^2^ g^−1^. FT-IR indicated successful polycondensation with the appearance of the C=N stretch at 1618 cm^−1^, while the stretches of the C=O and N-H monomers were attenuated. The surface area decreased with higher catalyst loading because of micropore blockage by the catalyst [107], yet all Nb_2_O_5_/COFs had narrow pore size distributions, therefore ruling out the reason being because of pore collapse (Figure 5c) [117]. SEM showed that most metal oxides were encapsulated within the COF matrix during the formation, rather than being surface localized. With the highest catalyst loading of 188.1 mol%, TGA analyses concluded 28.5% catalyst retention after purification, compared with an initial loading of 43.7% (Figure 5d). Kinetic experiments using Nb_2_O_5_ showed that 1 h was insufficient for high crystallinity and high-surface area COF products. Increasing the reaction time from 2 to 24 h resulted in a gradually more crystalline structure and a surface area jump from 680 to 1308 m^2^ g^−1^, while narrow pore size distributions were reached above 2 h. The increasing porosity was associated with the overtime reorganization of ordered sheets [118] or amorphous polymers into stacked structures [31]. In addition, various MOs (including nickel(II) oxide (NiO), manganese(IV) dioxide (MnO_2_), ruthenium(IV) oxide (RuO_2_), zinc(II) oxide (ZnO), lead(II) oxide (PbO), tellurium(IV) dioxide (TeO_2_), tin(IV) oxide (SnO_2_), manganese(III) oxide (Mn_2_O_3_), zirconium(IV) dioxide (ZrO_2_), and aluminum oxide (Al_2_O_3_)) produced highly crystalline COFs with high yields of 65–95% and high surface areas from 697 to 1644 m^2^ g^−1^, excluding ZrO_2_ which had yields with a lower surface area (Figure 5e). The authors explained that overall, the greater intrinsic Lewis acidity of the metal cation directly affects the yield, the surface area, the quality, and the crystallinity of the final COF products. The Lewis acidity of a metal oxide is affected by the MO crystal structure or phase and its water tolerance. Nb_2_O_5_, having a good water tolerance, leads to better catalytic performances (Figure 5f). The authors further show the generality of the Nb_2_O_5_ MO catalyst by substituting the monomer aldehyde group with (-F, -Br, -OCH_3_, -OH, -C_4_H_9_) and amines with a triazine backbone (Figure 6a). The resulting COFs had PXRD patterns and surface areas consistent with the literature on similarly reported COFs synthesized with acetic acid as the catalyst (Figure 6a–c). Importantly, the authors reported that when using Fe_3_O_4_ 30 nm nanoparticles (NPs) as the MO catalyst in the one-pot MO/COF composite synthesis, precipitation occurred instantly. The products had sharp PXRD peaks corresponding to the COF and the nanocomposite had an elevated surface area of 2196 m^2^ g^−1^. TGA showed that the MO retention was 9.65%. XPS did not detect the Fe_3_O_4_ catalyst because it was lodged deeper beneath the surface. TEM images further confirmed that the Fe_3_O_4_ NPs were monodispersed and embedded inside the large TAPB-OMePDA 2D sheets and uniformly distributed.

### 2.4. Metal Halides

**Figure 7:**
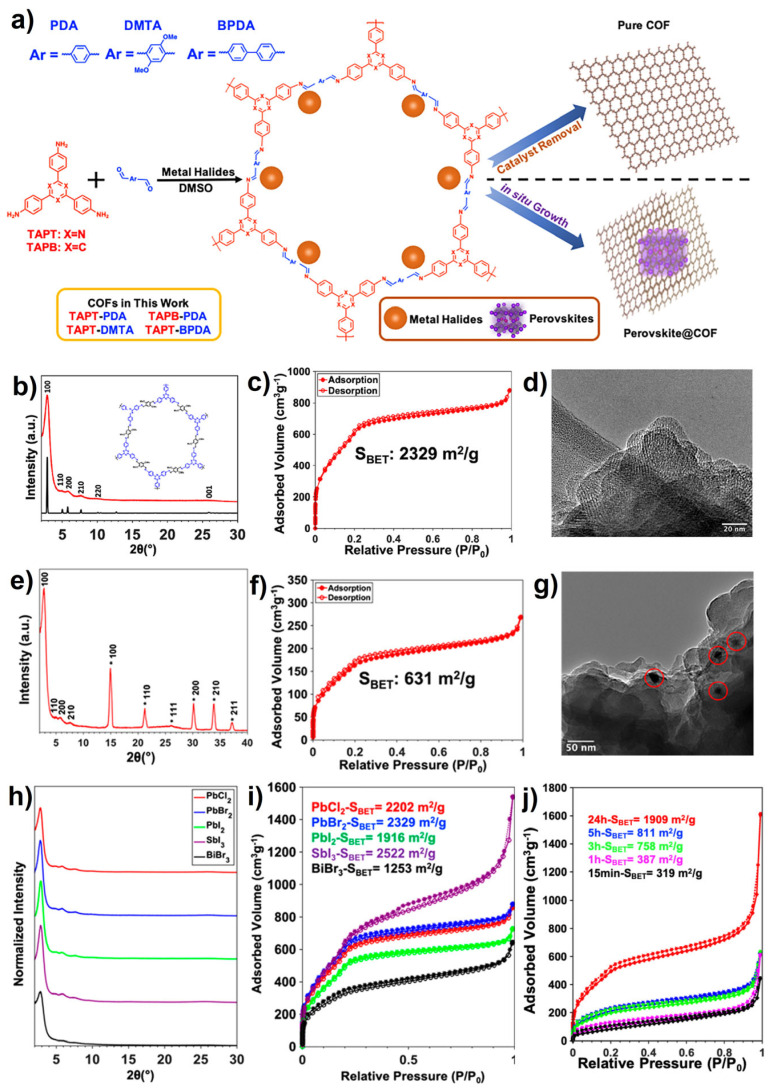
Synthesis of COFs and perovskite/COF nanocomposites in the presence of metal halides (**a**). PXRD patterns (**b**), BET surface area (**c**), and HRTEM image (**d**) of TAPT-DMTA COF synthesized with PbBr_2_ as a catalyst. PXRD patterns (**e**), BET surface area (**f**), and HRTEM image (**g**) of MAPbBr_3_@TAPT-DMTA COF composite. PXRD patterns (**h**) and BET surface areas (**i**) of TAPT-DMTA COFs synthesized with PbBr_2_, PbCl_2_, PbI_2_, SbI_3_, and BiBr_3_ as catalysts. BET surface areas of TAPT-DMTA COF in different reaction times (15 min to 24 h) (**j**). Reprinted with permission from ref. [107]. Copyright 2020, American Chemical Society.

An effective approach for the rapid synthesis of COFs and perovskite/COF nanocomposites is the use of metal halides. Sc(OTf)_3_, being a hard Lewis acid, was proven to be efficient in the rapid synthesis of COFs in ambient conditions even at trace loadings. The Group 14–15 metal halides also possess a Lewis acid characteristic and have been used as catalysts in many organic reactions [119,120]. Liu et al. are the first to have explored the in situ growth of methylammonium lead tribromide (MAPbBr_3_) perovskites with lead (Pb^2+^) and bromide (Br^−^) together as PbBr_2_ in the synthesis of MAPbBr_3_@TAPT-DMTA COF by the polycondensation of 1,3,5-tris(4-aminophenyl)triazine (TAPT) and 2,5-dimethoxyterephthaldehyde (DMTA) monomers (Figure 7a) [107]. Additionally, the authors explored the catalytic activity of COF production using antimony (Sb^3+^) and bismuth (Bi^3+^) as cations with chloride (Cl^−^), and iodide (I^−^) as anions. When employing PbBr_2_, a high crystallinity and a surface area of 2329 m^2^ g^−1^ were measured, which is among the highest ever reported for a 2D COF **(**Figure 7b–d) [59]. A control reaction between 4-methylaniline and benzaldehyde in the presence of only 0.056 equiv. of PbBr_2_ yielded 51% conversion into the imine product 1-phenyl-N-(*p*-tolyl)methanimine, which did not occur in the absence of PbBr_2_, confirming its role as the catalyst for transimination. The catalytic activities of PbCl_2_, PbBr_2_, PbI_2_, SbI_3_, and BiBr_3_ were also evaluated for the synthesis of TAPT-DMTA COF, producing highly crystalline materials with good yields of 73%, 80%, 79%, and 82% (Figure 7h). The surface areas ranged from 1253 to 2522 m^2^ g^−1^ (Figure 7i). The reactions catalyzed by three different metal halides (PbCl_2_, PbBr_2_, and PbI_2_) had the same rates (60–70 min) and yields (73–81%) with comparable BET surface areas (2202, 2329, and 1916 m^2^ g^−1^, respectively), suggesting that the counterion does not influence catalytic performance significantly. SbI_3_ afforded the fasted COF formation within 1 min with a high surface area of 2522 m^2^ g^−1^, which is probably associated with the stronger Lewis acid character compared with the other metal halides tested [121]. The COFs synthesized by the metal halides were found to bear greater surface areas compared with conventional methods. Versatility was demonstrated using SbI_3_ by synthesizing three other COFs (TAPT-PDA, TAPT-BPDA, and TAPB-PDA). In addition to having sharp crystalline peaks conforming to eclipsed stacking, all products had greater surface areas than those synthesized in conventional conditions [122]. As performed with PbBr_2_, SbI_3_’s catalytic activity was verified with a control experiment. Soaking the TAPT-DMTA COF made with PbBr_2_ into methylammonium bromide (MABr) alcoholic solution led to the growth of MAPbBr_3_ crystals, without affecting the chemical structure of the COF. The MAPbBr_3_ crystals were measured by HRTEM at about 14 nm and were identified by PXRD and XPS (Figure 7e–g). This COF composite showed a good BET surface area of 631 m^2^ g^−1^ compared with the bare COF (Figure 7f). Kinetic studies using SbI_3_ performed from 15 min to 24 h suggested more crystalline frameworks and a higher surface area with prolonged reaction time (Figure 7j). The presence of imines was detected as early as 15 min into the reaction by FT-IR. These observations were in accordance with previous mechanistic studies inferring the reorganization of amorphous structures into well-defined crystalline networks following a favorable dynamic imine exchange process, in this case catalyzed by SbI_3_ [31,46,79]. Seeing that most of the C=N stretch at 1625 cm^−1^ increased from 28% (at 15 min) to 78% (at 3 h), the authors assumed that the imine bond formation and exchange occurred simultaneously and prominently in the first 3 h of the reaction. Furthermore, the crystals showed notable stability in 75% humidity for 7 days and 2 months stored in ambient conditions, effectively demonstrating the protective role of COFs in preserving the normally labile perovskite crystals. Metal halides, particularly ZnCl_2_, have also proven effective in the synthesis of COFs featuring imide and olefin linkages [44,123,124], as well as triazine-linked covalent triazine frameworks (CTFs) [125,126,127,128,129,130]. The recent widespread adoption of metal halides in the synthesis of COFs with functional applications highlights their catalytic potency and versatility. This trend reflects the ability of metal halides to promote efficient bond formation under mild conditions, often leading to COFs with an enhanced crystallinity, porosity, and structural integrity suitable for a wide range of practical uses [131,132,133,134].

### 2.5. Metal Nitrates

**Figure 8:**
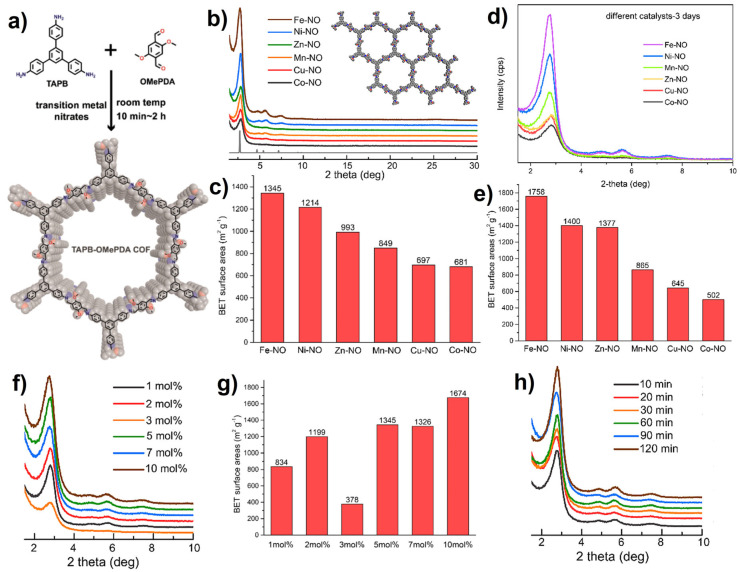
Synthesis of TAPB−OMePDA COF in the presence of metal nitrate (**a**). PXRD patterns (**b**) and BET surface areas (**c**) of TAPB−OMePDA COFs synthesized with six different metal nitrate (M-NO) catalysts. Kinetic comparison of the crystallinity (**d**) and surface area (**e**) of the M-NO catalyzed COFs. Effect of iron nitrate (Fe-NO) catalyst loading on COF crystallinity (**f**) and BET surface area (**g**). Effect of time on COF crystallinity in the presence of iron nitrate (Fe-NO) (**h**). Reprinted with permission from ref. [140]. Copyright 2021, American Chemical Society.

Although effective for catalyzing high-quality and rapid COF synthesis, scandium triflate (Sc(OTf)_3_) relies on an expensive rare earth element [135,136]. Solid-state catalysts, like metal oxides, have also been shown to be excellent for making MO/COF high-quality nanocomposites rapidly. But when the goal is to produce pure COFs, *p*-toluenesulfonic acid (PTSA) has been utilized as a solid-state catalyst in several studies [137,138,139]. However, large amounts of PTSA are required and recycling is not always an amenable option. Especially when considering that solid-state reactions accompany additional challenges with mixing and blending the starting materials or processing the final products, better options are desirable. Zhu et al. proposed using transition metal nitrates for the rapid synthesis of high-quality COFs with great yields [140] inspired by previous studies using transition metal nitrates (M-NO) for the practical synthesis of Schiff bases [141,142]. Six metal nitrates (Fe(NO_3_)_3_·9H_2_O, Ni(NO_3_)_2_·6H_2_O, Zn(NO_3_)_2_·6H_2_O, Mn(NO_3_)_2_·6H_2_O, Cu(NO_3_)_2_·6H_2_O, and Co(NO_3_)_2_·6H_2_O, denoted, respectively, as Fe-NO, Ni-NO, Zn-NO, Mn-NO, Cu-NO, and Co-NO were evaluated for the synthesis of a model system COF TAPB−OMePDA through the Schiff base polycondensation of 1,3,5-tris(4-aminophenyl)benzene (TAPB) and 2,5-dimethoxyterephthalaldehyde (OMePDA) (Figure 8a) [59,117,122]. For each test, 5 mol% catalyst was added relative to NH_2_ functional groups in ambient conditions, and the reaction time was 2 h, after which the powders were filtered and washed with solvent and supercritical CO_2_ prior to analysis. Standard techniques (FT-IR, ^1^H-^13^C CPMAS NMR, PXRD, and BET) confirmed TAPB−OMePDA COF formation by the appearance of the 1619 cm^−1^ C=N stretching peak following the loss of the 1685 cm^−1^ aldehyde stretching peak, imino carbon peaks at 155.8 and 150.4 ppm, and crystalline peaks in agreement with AA stacking and larger surface areas than 650 m^2^ g^−1^. Fe-NO-catalyzed COF had the most crystalline peak at 2*θ* = 2.7° and the greatest porosity (1345 m^2^ g^−1^), while Ni-NO also had appreciable porosity (1214 m^2^ g^−1^) (Figure 8b,c). Kinetic studies between 2 h and 3 days resulted in similar results between Fe-NO- and Ni-NO-catalyzed COF samples and indicated that 2 h was sufficient for crystalline structures with high surface areas and narrow pore size distributions for all M-NO evaluated (Figure 8d,e). Catalyst loading effects were prompted for Fe-NO ranging from 1 to 10 mol% relative to NH_2_ groups, corresponding to less than 3.5 mol% relative to TAPB monomers, for 2 h reaction times. Higher loadings (10, 7, and 5 mol%) produced the highest crystallinity, greater specific surface areas (1674, 1326, and 1345 m^2^ g^−1^ respectively) and narrower pore size distributions than other samples (Figure 8f,g). High-resolution XPS screening for residual iron in the final COF products was measured as trace amounts (0.02 atom% or 0.1 wt%) of total C 1s, N 1s, and O 1s signals. The catalyst Fe atoms were not found coordinated by nitrogen in the imine bond and neither was iron(III) reduced to metallic iron NPs during the synthesis. ICE-OES measurements on the final COFs resulted in the same 0.13 wt% Fe, demonstrating that the COFs were pure. Kinetic experiments from 10 min to 2 h using 10 mol% Fe-NO showed that crystallinity and porosity increased with time, but at 10 min all samples had very high surface areas of at least 1233 m^2^ g^−1^, increasing to 1674 m^2^ g^−1^ at 120 min (Figure 8h). The surface area results at 10 min reaction time were comparable to studies reporting the synthesis of TAPB−OMePDA COF using acetic acid as the catalyst at 120 °C for 3 days [143,144,145], and slightly lower than solvothermal conditions with nitrogen flow activation [59]. The versatility of this catalyst was verified by synthesizing previously reported COFs of different structural stabilities using acetic acid as the catalyst [117,145,146,147,148,149] and TAPB−4,4′-biphenyldicarboxaldehyde (BPDA) COF, which had only been synthesized with Sc(OTf)_3_ as the catalyst [79]. As anticipated, Fe-NO outperformed COF quality by producing highly crystalline TAPB−BPDA COF and TAPT−PDA COF, which could not be made using acetic acid as the catalyst. In addition, the BET surface areas were the highest reported for the COFs made with Fe-NO, with COF−V, TAPB−C8PDA COF, TAPT−PDA COF, and TAPT−OMePDA COF setting new records. Furthermore, no synthesis of 3D COFs and azine-linked ACOF-1 with 6 M acetic acid and Sc(OTf)_3_ were noted under the same conditions, whilst it was rapidly catalyzed at room temperature with 10 mol% Cu-NO. The only limitations were observed when trying to synthesize crystalline Py−1P imine COF using Py and PDA through a 4 + 2 construction strategy at room temperature due to poor solubility and 3D COFs COF-320 and COF-300. Despite Fe-NO being unable to synthesize 3D COFs, COF-320, and COF-300, 10 mol% of Cu-NO could produce these with high crystallinity in only 4 h at room temperature. Although unable to produce hydrazone COFs with either M-NO, using solvothermal methods with acetic acids or Sc(OTf)_2_, *β*-ketoenamine COFs with limited crystallinity were made with various M-NOs at room temperature for 6 h. An improvement in crystallinity occurred with an increased temperature of 120 °C. The authors recommend further mechanistic investigation, but proposed the Schiff base reaction with M-NOs to be related to the Brønsted acid nature of the transition metals in water or alcohol [150,151]. They suggested an increased solution acidity after complex formation between hydrated metals ions and water or 1-butanol, thus increasing the rate of the Schiff base reaction.

### 2.6. Heteropoly Acids

**Figure 9:**
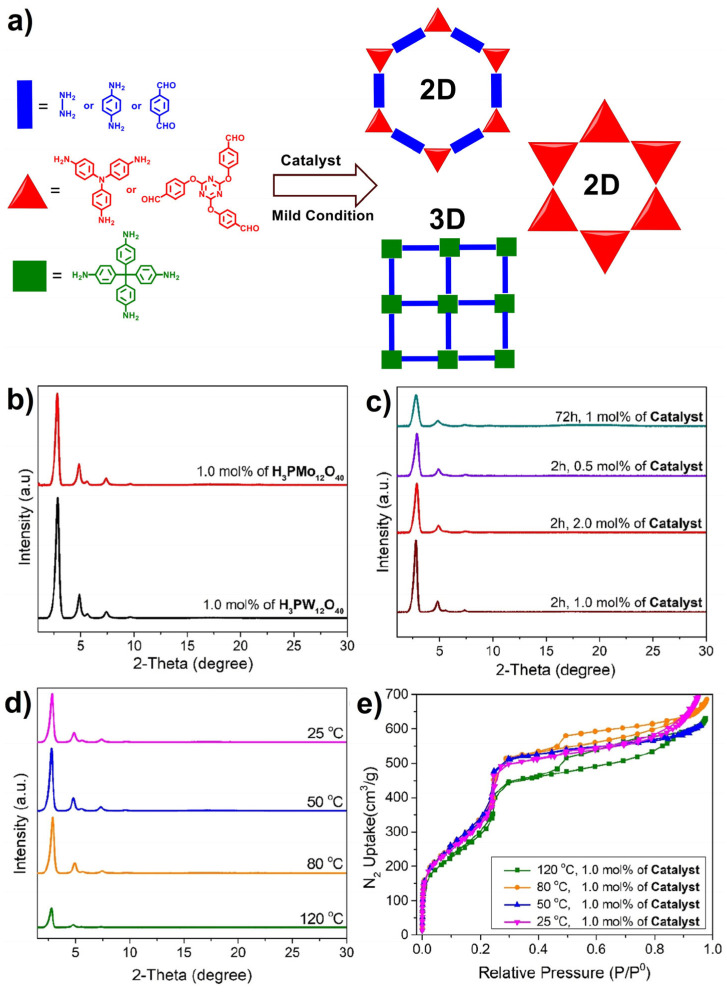
Catalytic synthesis of different 2D and 3D COFs using Keggin HPAs under mild conditions (**a**). PXRD patterns of TPT-PDA-COF synthesized with 1.0 mol% PTA and PMA HPA catalysts (**b**). Effect of reaction time on TPT-PDA-COF crystallinity using PTA HPA (**c**). Effect of reaction temperature on TPT-PDA-COF crystallinity using PTA HPA (**d**). Effect of reaction temperature on TPT-PDA-COF porosity using PTA HPA (**e**). Reprinted with permission from ref. [152]. Copyright 2023, American Chemical Society.

Recently we reported a scalable, economical, and effective approach for synthesizing imine-linked COFs using Keggin heteropoly acids (HPAs) [152]. HPAs are significantly stronger acids than sulfuric acid, an attribute previously shown to be effective in various organic reactions [153,154,155,156,157]. Their versatile solubility allows for their use in environmentally friendly solvents [158,159,160,161,162]. In this study, several 2D and 3D imine- and azide-linked COFs were successfully synthesized using the low-cost, commercially available, thermally stable, and highly oxidizing Keggin-type HPAs: phosphomolybdic acid (PMA, H_3_PMo_12_O_40_) and phosphotungstic acid (PTA, H_3_PW_12_O_40_) (Figure 9a) [163,164,165]. Initially, both catalysts were compared for the polycondensation of 2,4,6-tris(4-formylphenoxy)-1,3,5-triazine (TPT) and *p*-phenylenediamine (PDA) into TPT-PDA COF. X-ray diffraction analyses exhibited that highly crystalline products were obtained in the presence of 1.0 mol% of PMA and PTA, and after only 2 h at room temperature; however, PTA was able to catalyze the synthesis of TPT-PDA COF with a higher crystallinity (Figure 9b). Interestingly, longer reaction times led to a decrease in crystallinity, potentially due to the strong acidity of the catalyst adversely affecting the synthesized COFs (Figure 9c). Temperature had a minor effect on crystallinity; a slight increase from room temperature to 50 °C enhanced the diffraction peaks, but further increases were detrimental (Figure 9d). BET analyses supported these crystallinity results, with a maximal surface area observed at 50 °C (1277 m^2^ g^−1^) and a dramatic decrease at 120 °C (1079 m^2^ g^−1^). The surface area was comparable between 50 °C and room temperature (1180 m^2^ g^−1^) (Figure 9e). The pore sizes remained consistent across synthesis temperatures, ranging from 3.0 to 3.7 nm. To demonstrate universality, four other COFs, including those derived from TPT and hydrazine monohydrate (HZ), tris(4-aminophenyl)amine (TAPA) and terephthaldehyde (TPA), TAPA and TPT, and 3D COF 300 were prepared with a high crystallinity using PTA as the catalyst. Notably, this study marked the first reported synthesis of the imine-linked TAPA-TPT COF, which exhibited remarkable supercapacitor properties compared with ketoenamine-, boroxine-, and other imine-linked COFs [166,167,168].

## 3. Summary and Outlook

The advantages of various catalysts employed in the synthesis of imine-linked COFs, considering factors such as catalyst loading, reaction time, conditions, yield, crystallinity, and surface area, are summarized in Table 1, based on the representative studies discussed in this review. The synthesis of imine-linked COFs has historically been dominated by Brønsted acid catalysis, with acetic acid being a widely employed catalyst. This approach effectively promotes the reversible polycondensation reactions vital for achieving high crystallinity, but often necessitates harsh solvothermal conditions, thereby limiting scalability and environmental sustainability. Recent years have seen a significant shift, driven by the demand for greener, more efficient, and precisely controlled COF synthesis. This has spurred a comprehensive exploration of alternate catalyst strategies, moving beyond the conventional to unlock new synthetic pathways and expand the functional versatility of COFs. Key advancements in alternate catalysis include Lewis acid catalysis, metal oxides as heterogeneous catalysts, aqueous and green solvent systems, IL as reaction media and catalysts, and metal nitrates. Novel routes exploring electrochemically generated acids or enzyme catalysts and modulators are certainly in the scope of COF synthesis.

The diversification of catalyst strategies for COF synthesis is poised to revolutionize the field, driving both fundamental understanding and practical applications. A deeper understanding of the precise role of various Lewis acids is crucial. The unique environment provided by ILs and their potential for acid–base catalysis or template effects also warrants further mechanistic investigation. Developing new, highly efficient, and selective non-acidic catalysts remains a prime objective. Transitioning these advanced catalytic methods to large-scale, cost-effective industrial production is a major challenge. This necessitates the development of robust, recyclable, and easily separable catalytic systems. The push towards water-based, solvent-free, or low-solvent synthetic protocols will continue to intensify. The role of ILs as green reaction media or catalysts will also be a key focus. The ability to synthesize COFs directly onto substrates will unlock their potential in functional devices, sensors, membranes, and next-generation electronics. Combining different catalytic principles could lead to highly efficient and multifaceted approaches for COF formation with tailored properties.

In conclusion, the era of solely relying on Brønsted acid catalysis for COF synthesis is evolving. The exploration of diverse alternate catalytic strategies, encompassing a wide range of acetic acid, metal triflates, metal oxides, metal halides, metal nitrates, and the versatile use of ILs, is not just about finding new ways to make COFs, but about unlocking their full potential through precise control over their polycondensation, leading to superior materials for a wide array of applications in catalysis, energy, environmental remediation, and beyond. This ongoing research promises a vibrant future for COF chemistry.

## Figures and Tables

**Table 1:** Selected reports on catalytic methods for imine-linked COF synthesis.

Catalyst	Amount	Temperature/Pressure	Reaction Time	Yield	Crystallinity	Surface Area	Reference
**Acetic Acid**	6 M	80–120 °C/Autogeneous pressure	24–96 h	50–85%	Highly Crystalline	300–1500 m^2^ g^−1^	[32]
**Metal Triflate**	0.002 eq.	20 °C/Ambient pressure	1 min	95%		2175 m^2^ g^−1^	[79]
**Ionic Liquids**	100 μL	Ambient temperature and pressure	3 min	65%		517 m^2^ g^−1^	[91]
**Metal Oxides**	94 mol%	120 °C/Ambient pressure	3 days	65–95%		2405 m^2^ g^−1^	[116]
**Metal Halides**	0.056 eq.	90 °C/Ambient pressure	24 h	79%		2522 m^2^ g^−1^	[107]
**Metal Nitrates**	10 mol%	Ambient temperature and pressure	120 min	93.1%		1674 m^2^ g^−1^	[140]
**Heteropoly Acids**	1.0 mol%	50 °C/Ambient pressure	120 min	92%		1277 m^2^ g^−1^	[152]
